# Practical Departments

**Published:** 1903-06-20

**Authors:** 


### PRACTICAL DEPARTMENTS.

### HOSPITAL CONSTRUCTION.

INFIRMARY SECRETARY writes:—"My committee are anxious to ascertain the probable cost of building an up-to-date infirmary with from 30 to 40 beds, with operating-room, etc., and I venture to trouble you for an expression of opinion as to the cost per bed (purchase of site excluded). It has been suggested to have it one floor only in height, rooms for officials, nurses, and servants in the centre, and the wards on each side."

.·. If "Infirmary Secretary" obtains a copy of the third edition of Sir Henry Burdett's book, "Cottage Hospitals" (London, The Scientific Press), he will find on pages 278–9 a plan of a model pavillion hospital, with from 30 to 50 beds, which will give him practically all the particulars he wants. The total cost, as stated on page 282, is £5,250 for 48 beds, *i.e.*, £4,600 for building, and £650 for furnishing. That estimate was made some years ago, and it would be advisable to add 20s. per cent. to the figures, so as to be well within the limit of cost. It will be seen, on referring to the book, that the plan given provides that the wards shall be of one story only, with arrangements similar to those mentioned.—ED. THE HOSPITAL.

